# Supplementary material for: A look into the future of the COVID-19 pandemic in Europe: an expert consultation
Source: Lancet Reg Health Eur. 2021 Jul 30;8:100185. doi: 10.1016/j.lanepe.2021.100185 (PMC8321710; doi:10.1016/j.lanepe.2021.100185)
Supplement: Supplementary file 1 [file mmc1.docx]

# Looking into the future of the COVID-19 pandemic

We would like to hear your thoughts on the longterm perspective regarding COVID-19. This knowledge will enable the world to prepare for the expected challenges in time. Hence, in general:

- Please base your statements on evidence if possible; specify whether it is peer-reviewed publications, public health data and/or personal experience
- Please try to be specific (and quantitative if appropriate). Also make transparent what uncertainties there are.

Please structure your thoughts along the following five headings. Below the heading we have formulated some guiding questions (italic) for inspiration. You do not have to answer these questions; and feel welcome to make other relevant points as well.

# On general aspects of the COVID-19

- *What can cause further epidemic waves? (we are on purpose open here, from virus to behavioral changes)*
- *What would be required to achieve eradication of the virus?*
- *What degree of vaccination coverage / immunization do we need in the (global) population? When can we expect this level to be reached?*
- *How will the pandemic end?*

< your thoughts >

# What is the perspective for the coming summer?

- *What challenges do variants pose in this time period?*
- *Which preventive measures will remain necessary in this time period (e.g. non-pharmaceutical interventions)?*

< your thoughts >

# What is the perspective for the coming winter?

- *What challenges do variants pose in this time period?*
- *Which preventive measures will remain necessary in this time period (e.g. non-pharmaceutical interventions)?*

< your thoughts >

# What is the perspective for the coming 3-5 years?

- *What challenges do variants pose in this time period?*
- *Which preventive measures will remain necessary in this time period (e.g. non-pharmaceutical interventions)?*
- *What does an endemic situation look like (e.g. level of infections, burden to risk groups)?*

< your thoughts >

# Mitigating the effects of the COVID-19 pandemic

- What are potentially the 3 most important measures to take? - What are best (or worst) practice examples?
- What kind of surveillance data would one need for a better management of the future risks in this pandemic?

< your thoughts >
