# Supplementary material for: A look into the future of the COVID-19 pandemic in Europe: an expert consultation
Source: Lancet Reg Health Eur. 2021 Jul 30;8:100185. doi: 10.1016/j.lanepe.2021.100185 (PMC8321710; doi:10.1016/j.lanepe.2021.100185)
Supplement: Supplementary file 3 [file mmc3.docx]

# Looking into the future of the COVID-19 pandemic

We would like to hear your thoughts on the longterm perspective regarding COVID-19. This knowledge will enable the world to prepare for the expected challenges in time. Hence, in general:

- Please base your statements on evidence if possible; specify whether it is peer-reviewed publications, public health data and/or personal experience
- Please try to be specific (and quantitative if appropriate). Also make transparent what uncertainties there are.

Please structure your thoughts along the following five headings. Below the heading we have formulated some guiding questions (italic) for inspiration. You do not have to answer these questions; and feel welcome to make other relevant points as well.

# On general aspects of the COVID-19

- *In your country, what have been the most important social and economic risk factors in this pandemic? How have they been mitigated, if at all?*
- *How will public compliance with the rules develop? What will it depend on? Looking forward, how can compliance be improved?*
- *Will people be able to cope with continuing measures? What has to be done to ease the burden?*
- *What will be the impact of vaccination and of waning immunity?*

< your thoughts >

# What is the perspective for the coming summer?

- *What are the specific collateral effects of the pandemic in this time frame?*

< your thoughts >

# What is the perspective for the coming winter?

- *What are the specific collateral effects of the pandemic in this time frame?*

< your thoughts >

# What is the perspective for the coming 3-5 years?

- *What are the specific collateral effects of the pandemic in this time frame?*

< your thoughts >

# Mitigating the effects of the COVID-19 pandemic

- What are potentially the 3 most important measures to take? - What are best (or worst) practice examples?
- What kind of surveillance data would one need for a better management of the future risks in this pandemic?

< your thoughts >
