# Supplementary material for: A look into the future of the COVID-19 pandemic in Europe: an expert consultation
Source: Lancet Reg Health Eur. 2021 Jul 30;8:100185. doi: 10.1016/j.lanepe.2021.100185 (PMC8321710; doi:10.1016/j.lanepe.2021.100185)
Supplement: Supplementary file 4 [file mmc4.pdf]

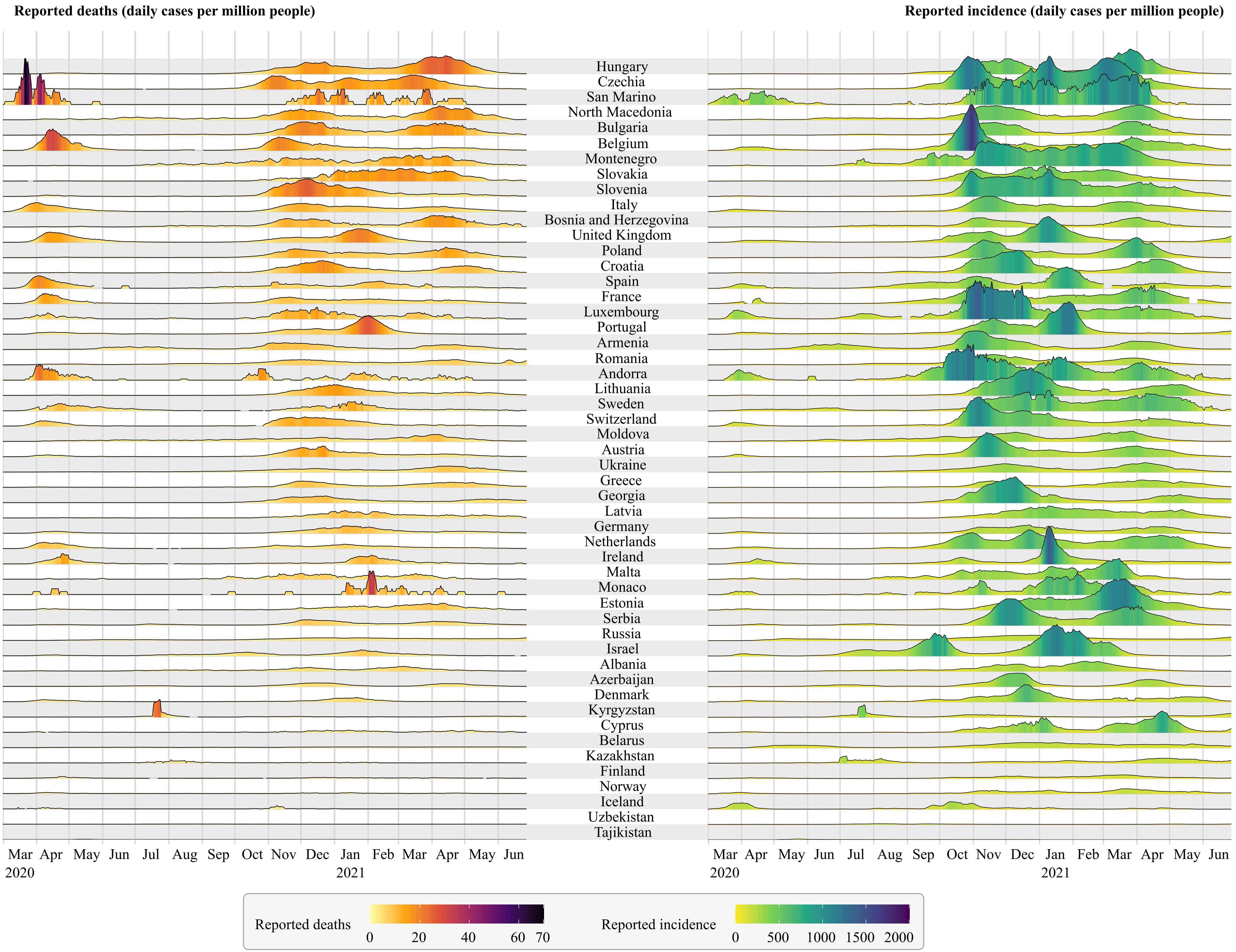

Reported deaths (daily cases per million people)
Reported incidence (daily cases per million people)
Hungary
Czechia
San Marino
North Macedonia
Bulgaria
Belgium
Montenegro
Slovakia
Slovenia
Italy
Bosnia and Herzegovina
United Kingdom
Poland
Croatia
Spain
France
Luxembourg
Portugal
Armenia
Romania
Andorra
Lithuania
Sweden
Switzerland
Moldova
Austria
Ukraine
Greece
Georgia
Latvia
Germany
Netherlands
Ireland
Malta
Monaco
Estonia
Serbia
Russia
Israel
Albania
Azerbaijan
Denmark
Kyrgyzstan
Cyprus
Belarus
Kazakhstan
Finland
Norway
Iceland
Uzbekistan
Tajikistan
Mar Apr May Jun Jul Aug Sep Oct Nov Dec Jan Feb Mar Apr May Jun
2020
2021
Mar Apr May Jun Jul Aug Sep Oct Nov Dec Jan Feb Mar Apr May Jun
2020
2021
Reported deaths
0 20 40 60 70
Reported incidence
0 500 1000 1500 2000
