# Supplementary figures and images for: A look into the future of the COVID-19 pandemic in Europe: an expert consultation
Source: Lancet Reg Health Eur. 2021 Jul 30;8:100185. doi: 10.1016/j.lanepe.2021.100185 (PMC8321710; doi:10.1016/j.lanepe.2021.100185)

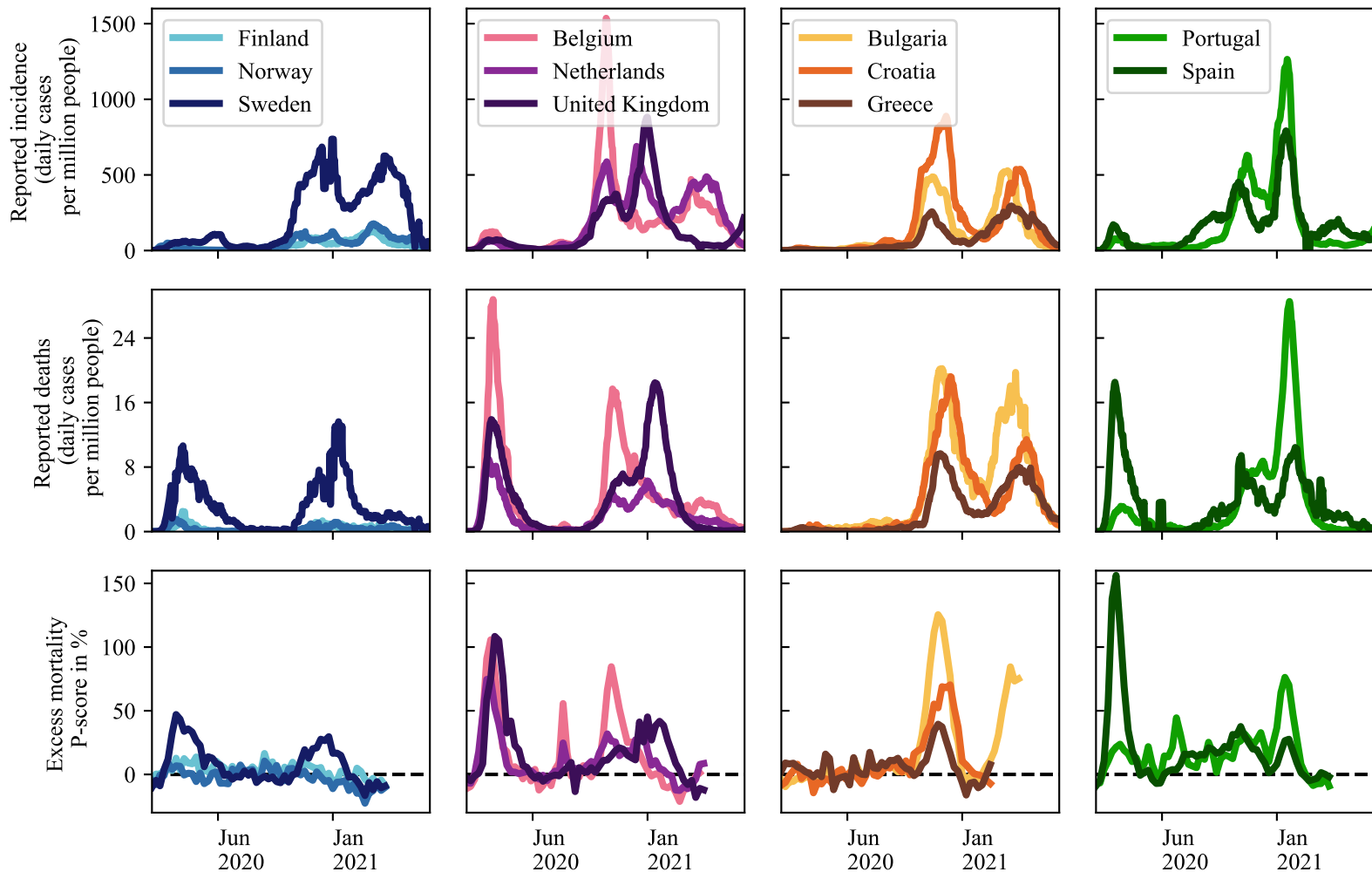

Supplement: Supplementary file 5 [file mmc5.pdf]

a

**Percent of population vaccinated with at least one dose as of June 26, 2021**

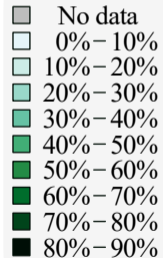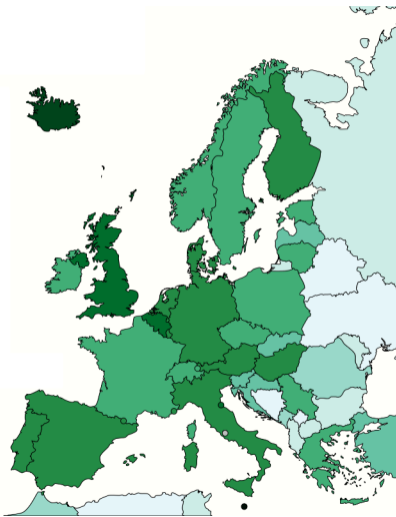

b

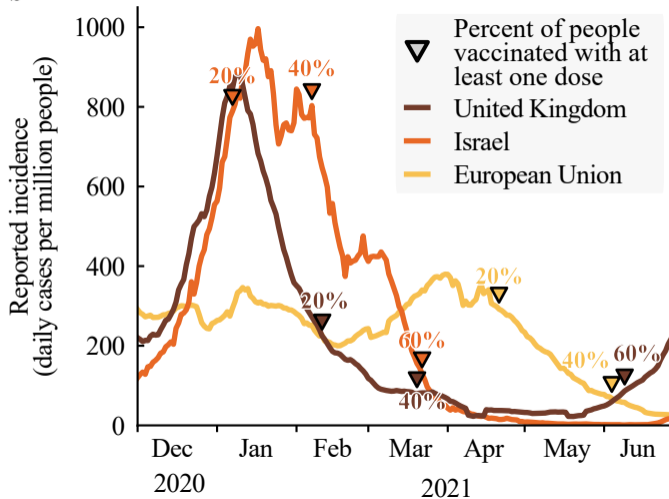

Supplement: Supplementary file 6 [file mmc6.pdf]
