# Supplementary material for: A look into the future of the COVID-19 pandemic in Europe: an expert consultation
Source: Lancet Reg Health Eur. 2021 Jul 30;8:100185. doi: 10.1016/j.lanepe.2021.100185 (PMC8321710; doi:10.1016/j.lanepe.2021.100185)
Supplement: Supplementary file 7 [file mmc7.docx]

# Looking into the future of the COVID-19 pandemic

We would like to hear your thoughts on the longterm perspective regarding COVID-19. This knowledge will enable the world to prepare for the expected challenges in time. Hence, in general:

- Please base your statements on evidence if possible; specify whether it is peer-reviewed publications, public health data and/or personal experience
- Please try to be specific (and quantitative if appropriate). Also make transparent what uncertainties there are.

Please structure your thoughts along the following five headings. Below the heading we have formulated some guiding questions (italic) for inspiration. You do not have to answer these questions; and feel welcome to make other relevant points as well.

# On general aspects of the COVID-19

- *How long does immunity last (natural infection / vaccine)?*
- *Will re-infections be less or more severe?*
- *What kind of variants will develop, with which probability, and what drives their development?*
- *How should one prevent and potentially face escape-variants?*
- *What role do animal reservoirs play?*
- *Will or should children be vaccinated? Why?*
- *How will the pandemic end?*

< your thoughts >

# What is the perspective for the coming summer?

- *What is specifically to be expected in this time period?*
- *What variants will develop in this time period, and can one quantitatively predict their impact (e.g. contagiousness, deadliness, immune escape)?*

< your thoughts >

# What is the perspective for the coming winter?

- *What is specifically to be expected in this time period?*
- *What variants will develop in this time period, and can one quantitatively predict their impact (e.g. contagiousness, deadliness, immune escape)?*

< your thoughts >

# What is the perspective for the coming 3-5 years?

- *What is specifically to be expected in this time period?*
- *What variants will develop in this time period, and can one quantitatively predict their impact (e.g. contagiousness, deadliness, immune escape)?*

< your thoughts >

# Mitigating the effects of the COVID-19 pandemic

- What are potentially the 3 most important measures to take? - What are best (or worst) practice examples?
- What kind of surveillance data would one need for a better management of the future risks in this pandemic?

< your thoughts >
