# Supplementary material for: A look into the future of the COVID-19 pandemic in Europe: an expert consultation
Source: Lancet Reg Health Eur. 2021 Jul 30;8:100185. doi: 10.1016/j.lanepe.2021.100185 (PMC8321710; doi:10.1016/j.lanepe.2021.100185)
Supplement: Supplementary file 8 [file mmc8.docx]

# Caption for supplementary material

methods.docx: The methodology underlying the systematic expert consultation and writing of the text.

questions.zip: File containing the four initial questionnaires for the collaborators.
