# Supplementary material for: A look into the future of the COVID-19 pandemic in Europe: an expert consultation
Source: Lancet Reg Health Eur. 2021 Jul 30;8:100185. doi: 10.1016/j.lanepe.2021.100185 (PMC8321710; doi:10.1016/j.lanepe.2021.100185)
Supplement: Supplementary file 9 [file mmc9.docx]

## Methods

The methodology to generate and compile the content of this manuscript is inspired by the Delphi method.^1^ It describes a process for facilitating discussions or forecasts by groups of people. Key characteristics of this method are that (a) the participants do not communicate directly with each other while formulating their opinions and that (b) the synthesis of the content is moderated by designated facilitators. One advantage of the Delphi method is that it reduces some common group biases.^2^ The facilitators in this case were Viola Priesemann, Emil Iftekhar, Sebastian Mohr, and Simon Bauer (all affiliated with the Max Planck Institute for Dynamics and Self-Organization, Göttingen, Germany). The precise individual steps of the process were the following:

### **1. Formulating instructions for the collaborators**

The facilitators drafted general instructions and sets of guiding questions to send out to the collaborators (see supplementary material “questions.zip”). The guiding questions were formulated with the aim of serving as inspiration only. The facilitators prepared four different sets of questions, structured along five general headings:

1. On general aspects of COVID-19
2. What is the perspective for the coming summer?
3. What is the perspective for the coming winter?
4. What is the perspective for the coming 3-5 years?
5. Mitigating the effects of the COVID-19 pandemic

Each questionnaire included general questions and questions regarding a specific field of research: epidemiology and dynamics, public health, social sciences, and virology. The initial outline of this methodology and the guiding questions were refined with the help of some experts (see [Table 1](#kix.94vaul1icfxv)) and finalized by the facilitators.

| **Name** | **Country of affiliation** | **Field of expertise** |
| --- | --- | --- |
| Peter Klimek | Austria | Mathematical modeling |
| Barbara Prainsack | Austria | Political science, bioethics |
| Eva Schernhammer | Austria | Public health, medicine |
| Carlos Martins | Portugal | Family medicine |
| Mirjam Kretzschmar | The Netherlands | Infectious disease dynamics |

Table 1: List of experts contributing to the design of the methodology and the formulation of the instructions for the rest of the collaborators.

### **2. Choosing the collaborators**

The facilitators chose the collaborators for the Delphi study such that all four selected fields were about evenly represented and that as many European countries as possible were represented. The prospective collaborators were largely selected from the facilitators’ existing professional networks or based on recommendations by colleagues. As a result, 98 experts from 31 countries were contacted. As the invitations were via email, it is possible that the invitations did not reach a few of them due to spam filtering. Of the experts that were contacted, 30 people from 17 European countries replied and subsequently contributed to the study as collaborators (see [Table 2](#kix.rl567bezd201) and Figure 4). Note that three of the collaborators were also involved in refining the questionnaires and methods. Even though this is not a strict division of labor between facilitators and collaborators, we deem the thereby-introduced influence on the content as negligible.


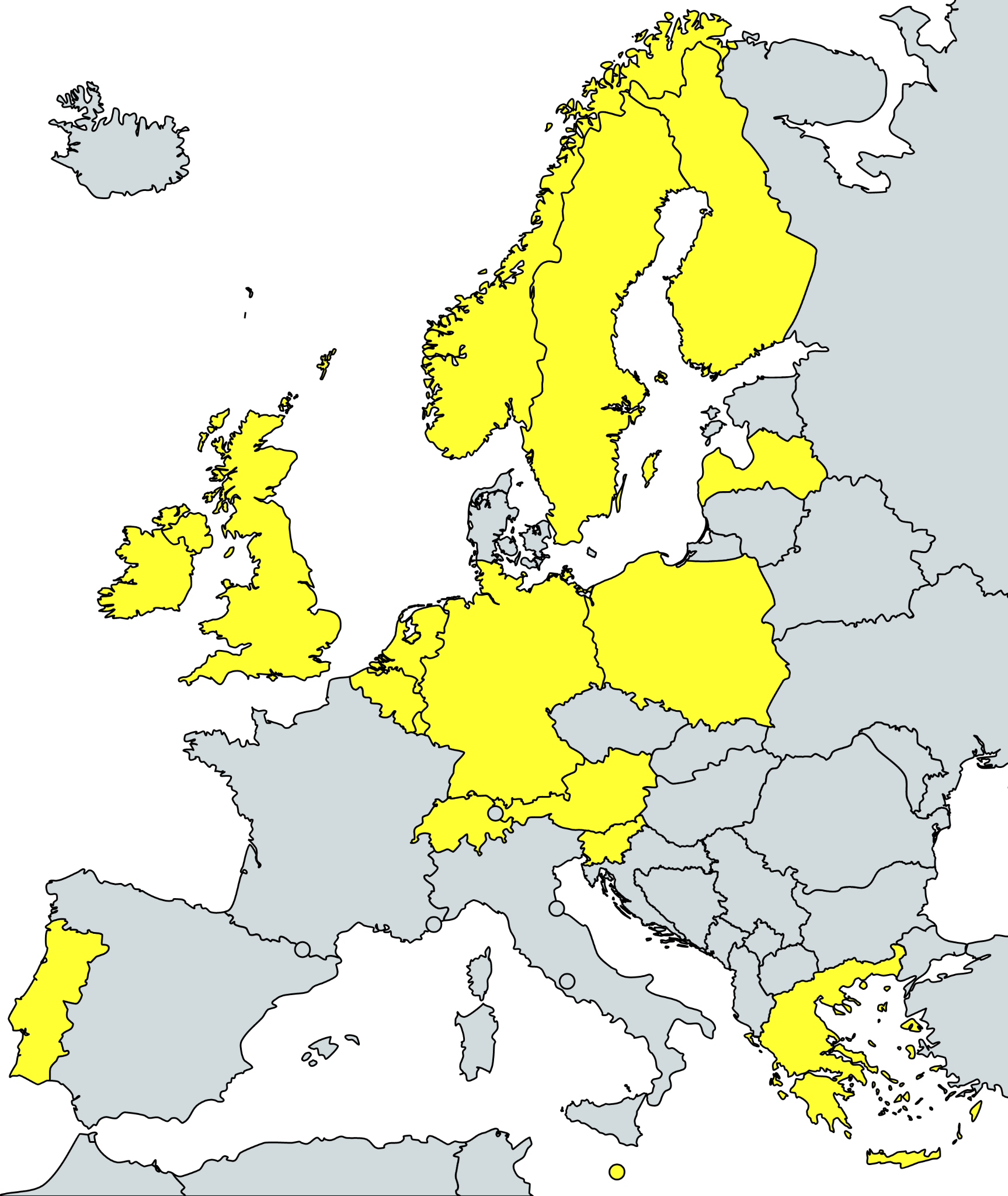


Figure 4: Country affiliations of the collaborators (marked in yellow).

| **Name** | **Country of affiliation** | **Questionnaire assigned by facilitators** |
| --- | --- | --- |
| Thomas Czypionka | Austria | Social sciences |
| Peter Klimek | Austria | Epidemiology and dynamics |
| Eva Schernhammer | Austria | Epidemiology and dynamics |
| Peter Willeit | Austria | Epidemiology and dynamics |
| Philippe Beutels | Belgium | Epidemiology and dynamics |
| Steven Van Gucht | Belgium | Virology |
| Pirta Hotulainen | Finland | Epidemiology and dynamics |
| Eva Grill | Germany | Public health, virology |
| Gérard Krause | Germany | Epidemiology and dynamics |
| Armin Nassehi | Germany | Social sciences |
| André Calero Valdez | Germany | Social sciences |
| Elena Petelos | Greece | Public health |
| Sotirios Tsiodras | Greece | Virology |
| Anthony Staines | Ireland | Public health |
| Uga Dumpis | Latvia | Virology |
| Rudi Balling | Luxembourg | Virology |
| Enrico Glaab | Luxembourg | Virology |
| Sarah Cuschieri | Malta | Public health |
| Jenny Krutzinna | Norway | Social sciences |
| Tyll Krüger | Poland | Epidemiology and dynamics |
| Ewa Szczurek | Poland | Epidemiology and dynamics |
| Helena Machado | Portugal | Social sciences |
| Matjaž Perc | Slovenia | Epidemiology and dynamics |
| Claudia Hanson | Sweden | Public health |
| Joacim Rocklöv | Sweden | Epidemiology and dynamics |
| Nicola Low | Switzerland | Epidemiology and dynamics |
| Mirjam Kretzschmar | The Netherlands | Epidemiology and dynamics |
| Sebastian Funk | UK | Epidemiology and dynamics |
| Martin McKee | UK | Public health |
| Martyn Pickersgill | UK | Social sciences |

Table 2: List of collaborators participating in the Delphi-survey and their respective countries of affiliation. The third column indicates which of the four field-specific questionnaires the collaborator received from the facilitators.

### **3. Writing the document**

All collaborators (see [Table 2](#kix.rl567bezd201)) were invited to take part in this Delphi forecast on the long-term perspective regarding the COVID-19 pandemic in Europe. They were provided (a) an explanation of the steps of the process, (b) the general instructions and (c) at least one set of questions related to their field of expertise. The collaborators were asked to send their input separately to facilitators and to not communicate with each other. Though provided with guiding questions, answering them was communicated to be optional and the collaborators were welcomed to make further points.

After receiving all inputs, the facilitators summarised and synthesized the inputs into one document. The resulting document was sent out to the collaborators, along with all single replies of the collaborators. This was followed by two rounds of revisions; in each round, (1) the collaborators were asked to send their comment separately to the facilitators, (2) the facilitators incorporated the comments, and (3) presented the updated document to the collaborators. The contributions of the collaborators included references to pertinent scientific literature to support and evidence their statements. In the second step of incorporating the collaborators’ comments, the facilitators added additional references to some statements in the manuscript. These additional references were then accepted, amended, or corrected by the collaborators. As a last step, the collaborators were able to suggest minor corrections in a shared online document and were asked to confirm their authorship after the finalization of the draft. The process of writing the document started on March 8, 2021, and ended on May 6, 2021.

**References**

1. Brown BB. Delphi process: a methodology used for the elicitation of opinions of experts. Rand Corp Santa Monica CA; 1968.

2. Linstone HA, Turoff M. The delphi method. Reading, MA: Addison-Wesley; 1975.
